# Healthcare professionals’ views of physiotherapy after cardiac surgery in children with congenital heart disease: a UK and Ireland survey

**DOI:** 10.1136/bmjopen-2024-097314

**Published:** 2025-11-12

**Authors:** Stephanie L Clarke, Emma Shkurka, Julie C Menzies, Nigel E Drury

**Affiliations:** 1 Department of Physiotherapy, Birmingham Children’s Hospital, Birmingham, UK; 2 Department of Physiotherapy, Great Ormond Street Hospital for Children, London, UK; 3 Department of Paediatric Critical Care, Bristol Royal Hospital for Children, Bristol, UK; 4 Department of Cardiovascular Sciences, University of Birmingham, Birmingham, UK; 5 Department of Paediatric Cardiac Surgery, Birmingham Children’s Hospital, Birmingham, UK

**Keywords:** Congenital heart disease, Cardiac surgery, Physical Therapy Modalities, Physical Fitness, Developmental neurology & neurodisability

## Abstract

**Abstract:**

**Objectives:**

To understand healthcare professionals’ views on current physiotherapy service provision in children with congenital heart disease (CHD), how physiotherapy could be better used post-cardiac surgery and perceived barriers to service expansion.

**Design:**

Cross-sectional survey using a one-off self-completed online questionnaire, with open and closed questions, in June–August 2024.

**Setting:**

Each of the 12 level 1 paediatric cardiac surgical centres in the UK National Health Service and Children’s Health Ireland.

**Participants:**

Healthcare professionals providing clinical care to children undergoing cardiac surgery.

**Results:**

80 responses were obtained, with at least one response from each centre. Healthcare professionals conduct motor, developmental and functional evaluations across all age groups, with referrals to physiotherapy primarily based on physical examination (39, 87%). They expressed dissatisfaction with community physiotherapy services (64, 81%) compared with inpatient services (29, 36%), although they indicated that expanding services would positively impact patients and families. There is a lack of consensus regarding intervention frequency, duration and which patient groups should be prioritised. Respondents identified a lack of funding as the primary barrier to service expansion (76, 95%). Reported barriers for families included volume of medical appointments (69, 86%), transportation (66, 83%) and finances (62, 78%).

**Conclusions:**

Healthcare professionals appreciate the positive impact physiotherapy can have on post-surgical management of children with CHD. The importance of expanding services was emphasised. However, to effectively support clinical practice, it is crucial to understand which patient groups should be prioritised and at what stage, as well as determining the optimal amount of physiotherapy that positively impacts patient outcomes.

**STRENGTHS AND LIMITATIONS OF THIS STUDY**

- This survey explores the provision of physiotherapy services to children undergoing surgery for congenital heart disease in the UK and Ireland.
- The use of quantitative and qualitative data allowed for deeper insights into the views of healthcare professionals caring for children with congenital heart disease.
- By sampling only healthcare professionals from level 1 paediatric cardiac surgical centres, the findings may not be representative of the wider population, which limits generalisability.
- The recruitment strategy also resulted in under-representation of specific professional groups, limiting subgroup analyses.

## Introduction

 Congenital heart disease (CHD) is one of the most common congenital malformations representing 57.1 per 10 000 live births in England.^1 2^ With advancements in surgical techniques and perioperative care, early mortality^3^ and late survival have increased, with 97% of children diagnosed with CHD living to adulthood.^4^ As more children survive, there is growing awareness of the impact of CHD on developmental outcomes, physical function and quality of life (QOL).

Children with CHD display delayed motor development and functional impairment from infancy through to adolescence.^5^ This is due to altered foetal brain development, perioperative brain injury and post-surgical complications. One third of children with CHD require cardiac surgery or other interventions in the first year of life, and many require further operations during childhood and beyond.^6^ Negative developmental and functional outcomes linked to cardiac surgery include prolonged bypass time, increased hospital length of stay and postoperative adverse events.^7^ In the first year of life, mild to severe motor developmental delay is present in up to 55% of infants with CHD. Throughout childhood, impaired function has been illustrated in up to 60% of toddlers, 48% of preschool children and 46% of school-aged children.^5 8 9^ In the recent James Lind Alliance Priority Setting Partnership, reducing the effects of CHD and interventions on brain development and improving the QOL of children living with CHD were identified as national priorities for research.^10^

The American Heart Association (AHA) and Cardiac Neurodevelopmental Outcome Collaborative released statements recommending strategies to systematically evaluate neurodevelopment and function in children with CHD. They support the use of routine developmental and functional screening, and referral to the multidisciplinary team including physiotherapists.^11^ The potential role of physiotherapists in post-surgical rehabilitative pathways in children with CHD has also been illustrated within the literature.^12^

The value of physiotherapeutic rehabilitation in children with CHD has been evaluated in multiple small studies, which demonstrate a positive impact on motor function, strength and developmental milestones.^13 14^ However, due to the heterogeneity of these studies (variable patient populations, interventions and study designs), they do not provide an adequate evidence base on which physiotherapy service delivery can be established for the postoperative management of children with CHD. Thus, further research is required to build a comprehensive body of evidence on which physiotherapeutic programmes for children with CHD can be based.

Future intervention development should be cognisant of current clinical practices, potential barriers to the implementation of physiotherapy and the key research priority areas. Therefore, gaining insights from key stakeholders, especially healthcare professionals (HCPs), will enhance the likelihood of achieving clinically viable outcomes that are valued in practice. There is minimal published literature conveying the views of HCPs on physiotherapeutic interventions and service provision in children with CHD. A 2002 survey of UK-based paediatric cardiologists reported that only 18% of respondents felt that rehabilitation services for children with CHD met the needs of the patient and their family.^15^ This survey highlights the value of using HCPs’ clinical experience to ensure proposed interventions are acceptable and feasible.^16^ This study aimed to understand HCPs’ views on current physiotherapy service provision, how physiotherapy could be better used in children with CHD post-cardiac surgery and perceived barriers to service expansion. Conclusions drawn from this study may support the development of future interventions, highlight priority areas for service expansion and categorise barriers to service delivery.

### Research questions

1. How do HCPs view their role in the evaluation and management of motor delay or impaired physical function in children with CHD?
2. How do HCPs view current physiotherapy services for children with CHD?
3. What are HCPs’ opinions on how physiotherapy services could be better used in children with CHD and what are the barriers to service provision?

## Methods

### Patient and public involvement

The four domains of the survey were shaped based on insights and feedback shared by six parents whose child has a diagnosis of CHD, and who are members of a local charity *Young at Heart*. The patient and public involvement for this study was gathered in conjunction with feedback for a qualitative study that covers the same domains, using a mix of focus groups and asynchronous input.

### Design

This study involved a cross-sectional survey using a one-off self-completed online questionnaire. The Consensus-Based Checklist for Reporting of Survey Studies was used in writing this report.^17^

### Study population

All HCPs providing clinical care to children undergoing cardiac surgery working within level 1 paediatric cardiac surgical centres in the UK National Health Service (NHS) and Children’s Health Ireland were included.

### Recruitment

HCPs were recruited via professional membership organisations, social media and snowball sampling.^18^ The British Congenital Cardiac Association, Association of Chartered Physiotherapists in Respiratory Care, the Congenital Cardiac Nurse Association and Extra Corporeal Membrane Oxygenation paediatric specialist physiotherapy interest groups disseminated a survey link to their members. Participants were asked to share the survey with other individuals who met the inclusion criteria. The survey was open for an 8-week period from June to August 2024.

### Sample size

This was a non-experimental study; thus, a null hypothesis was not established, data were not evaluated using inferential statistical analysis, and a sample size calculation was not performed. A minimum of one response from each of the 12 level 1 paediatric cardiac surgical centres was sought to ensure geographical representation of service provision across the UK and Ireland.

### Data collection

There were no standardised validated tools available that met the needs of this study. Survey questions were developed from published literature (online supplemental material 1). Six professionals were asked to pre-test the survey and provide feedback regarding face and content validity. These included advanced physiotherapists, a consultant paediatric intensivist and paediatric intensive care nurse, all of whom have experience working with children with CHD. Amendments were made to improve question clarity, increase free-text box size and question order. The introductory paragraph was also expanded to improve the transparency of the study aims. Details of the face and content validation process are summarised in online supplemental material 2.

The survey comprised 15 questions across four domains: demographic information, usual practice, current service provision and service expansion (online supplemental material 3). The survey questions were filtered to reflect the respondents' professional roles. 10 questions were open to all professional groups, 4 to physiotherapists only and 1 to non-physiotherapist HCPs. Included were a combination of multiple choice, dichotomous and a single open question. Based on pre-testing, the survey took 10 min to complete. The survey was conducted online using a one-off self-reported web-based questionnaire. The survey was hosted, and the study data collected and managed using Research Electronic Data Capture tool (see acknowledgements).^19^

### Data analysis

The data collected was downloaded as an Excel spreadsheet. Descriptive statistics including frequency counts and percentages were used for dichotomous data and Likert scale data. Quantitative data were displayed using bar charts. Content analysis of qualitative data (question 13) was completed by two researchers independently using MaxQDA software.^20^ The free text was systematically coded by each researcher to highlight key words. Clusters of similar codes were combined which shared similar themes to form categories and subcategories. The categorisation and subcategorisation of the codes were subsequently compared, and a consensus was reached. Subcategories included reference to the frequency and percentage of people who shared thoughts to provide an indicator of the degree of consensus.

## Results

A total of 80 valid responses were obtained from 34 (43%) physiotherapists and 46 (57%) other HCPs (table 1) with at least one response from each level 1 paediatric cardiac surgical centre in the UK and Ireland. One responsevey was excluded, as the respondent was not based at a level 1 cardiac centre. Four questions were not completed in full by 11 (14%) participants (questions 5, 6, 9 and 11).

**Table 1:** Survey responses by professional group

Respondents, n (%)	
Physiotherapists	34 (43)
Other healthcare professionals	46 (58)
Cardiac nurse specialist	13 (17)
Paediatric cardiologist	9 (11)
Occupational therapist	7 (9)
Paediatric cardiothoracic surgeon	5 (6)
Consultant intensivist	5 (6)
Advanced clinical practitioners	4 (5)
Clinical scientist	1 (1)
Cardiac staff nurse	1 (1)
Speech and language therapist	1 (1)

### Usual practice

**Figure 1:**
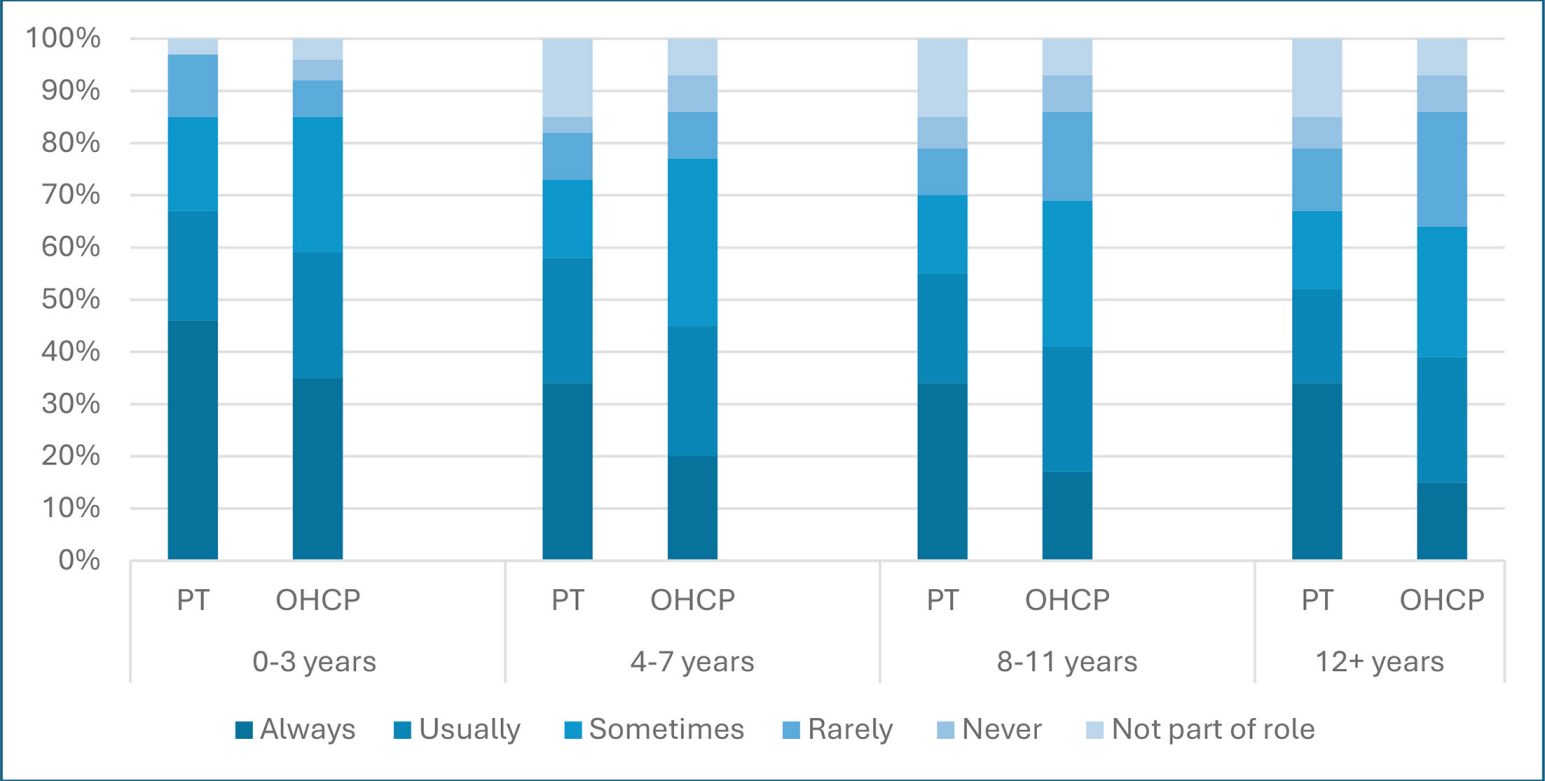
Frequency of developmental and functional discussions with parents following cardiac surgery.

Motor development or functional abilities were most frequently discussed with parents following cardiac surgery in children aged 0–3 years with 50 (63%) respondents stating they ‘always’ or ‘usually’ discussed this, decreasing to 41 (51%) in children aged 4–7 years, and to less than 50% in children over the age of 8 years (8–11 years 38 (48%), 12+ years 36 (45%)), figure 1. Other HCPs were asked what would trigger a referral to physiotherapy. ‘Following physical examination’ (40, 87%) was the most frequently selected answer, followed by ‘parental concern’ (36, 78%) then ‘meeting high risk criteria’ (31, 67%). 30 (65%) respondents stated that all three criteria would prompt a referral to physiotherapy.

### Current service provision

33 (72%) other HCP respondents reported they were ‘very satisfied’ or ‘satisfied’ with inpatient services. Physiotherapists were split on their satisfaction with inpatient services, with an equal number ‘very satisfied’ or ‘satisfied’ (16, 47%) compared with those who were ‘dissatisfied’ or ‘very dissatisfied’ (16, 47%).

18 (39%) other HCPs reported they were ‘dissatisfied’ or ‘very dissatisfied’ with outpatient and community physiotherapy services, compared with 25 (74%) physiotherapists (table 2).

**Table 2:** Satisfaction with inpatient and outpatient/community physiotherapy service provision

Respondents, n (%)		Very satisfied	Satisfied	Neither satisfied nor dissatisfied	Dissatisfied	Very dissatisfied	No opinion
Inpatient service provision	PT	5 (15)	11 (32)	2 (6)	12 (35)	4 (12)	0 (0)
	OHCPs	20 (44)	13 (28)	4 (9)	8 (17)	1 (2)	0 (0)
Community/outpatient service provision	PT	0 (0)	4 (11)	3 (9)	17 (50)	8 (24)	2 (6)
	OHCPs	1 (2)	9 (20)	7 (15)	14 (30)	4 (9)	11 (24)

OHCPs, other healthcare professionals; PT, physiotherapists.

### Service expansion

33 (97%) physiotherapists and 39 (85%) other HCP respondents reported that inpatient service expansion would improve care, with a similar finding for outpatient services: 34 (100%) physiotherapists and 45 (98%) other HCP respondents. Routine developmental or functional screening during cardiologist appointments was reported to benefit patients and families by 33 (97%) physiotherapists and 40 (87%) other HCP respondents.

### Physiotherapist reported service priorities

**Figure 2:**
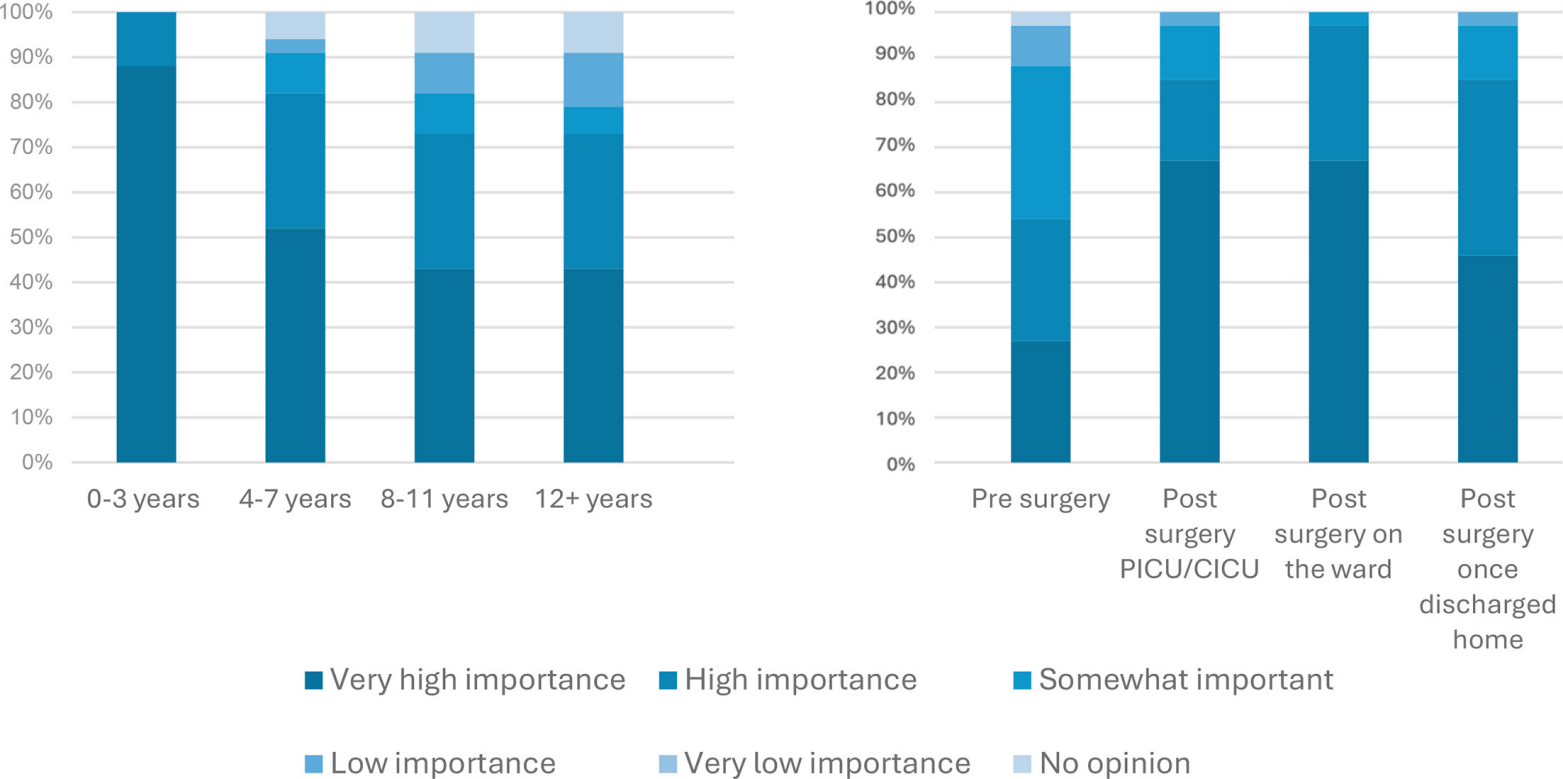
Importance of physiotherapy (**a**) at different ages and (**b**) at different stages of the patient journey. CICU, cardiac intensive care unit; PICU, paediatric intensive care unit.

Physiotherapists reported interventions were ‘high’ or ‘very high’ importance in children aged 0–3 years (33, 100%), with fewer respondents attaching this level of importance as children became older: 27 (82%) in children aged 4–7 years and 4 (73%) in children over 8 years of age. They identified post-surgery on the ward as the most important time for interventions (32, 97%), ahead of on paediatric intensive care unit/cardiac intensive care unit or once discharged home (28, 85%), as shown in figure 2. They also reported it was ‘more’ or ‘most important’ to deliver outpatient care within the patient’s home (25, 81%). Care delivered within the hospital (14, 42%), community centres (13/30, 43%) and in school/nurseries (10/30, 31%) all had similar importance. Virtual appointments were reported to be ‘least important’ or ‘less important’ (30, 88%). 21 (64%) physiotherapists reported that community/outpatient physiotherapists should have specialist knowledge of CHD, whereas 7 (21%) did not think this was required and 5 (15%) were unsure.

**Figure 3:**
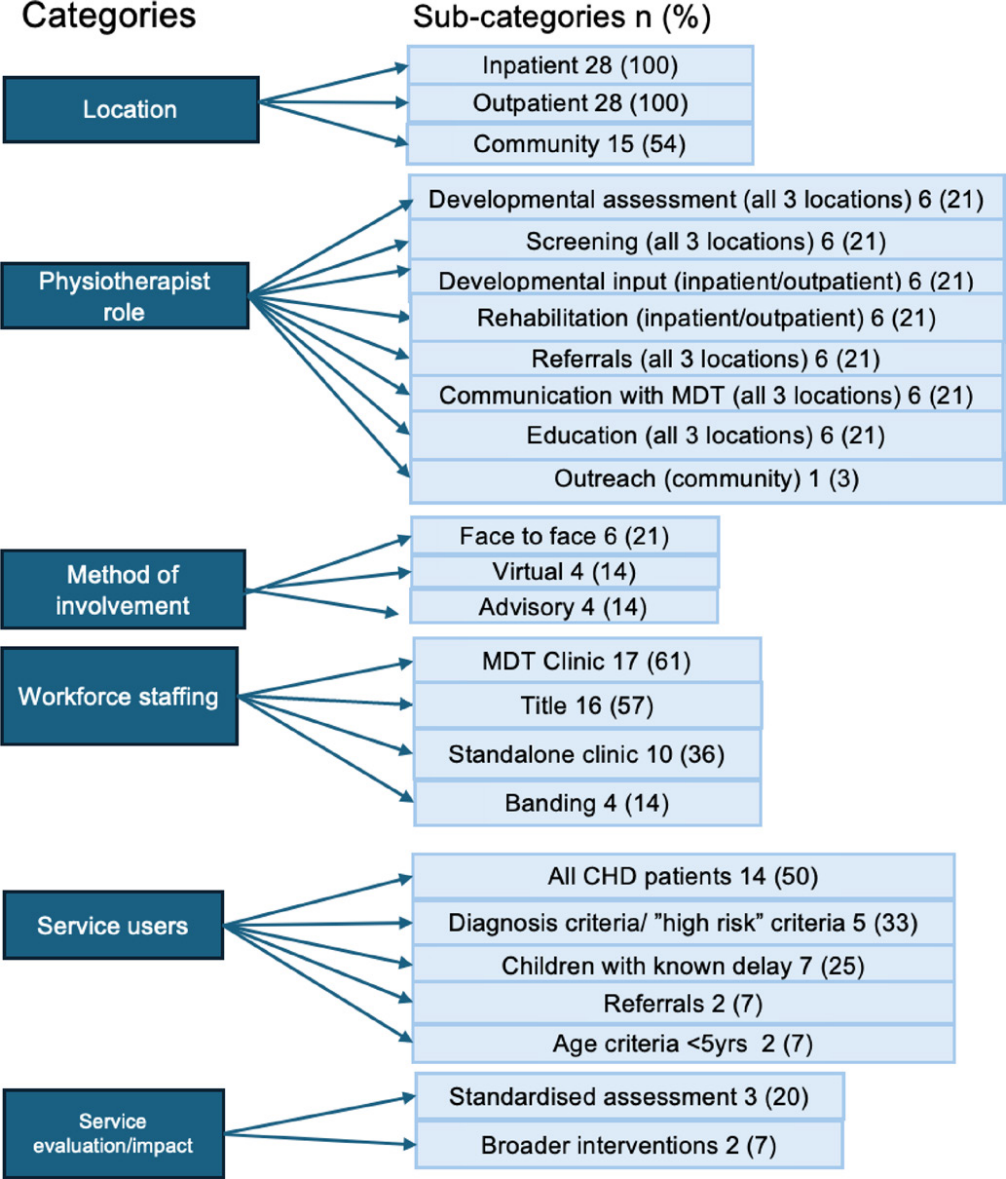
Content analysis of respondents’ ‘ideal’ physiotherapy service for children with congenital heart disease. CHD, congenital heart disease; MDT, multidisciplinary team.

Content analysis of the free-text question on what an ‘ideal’ physiotherapy service would resemble identified six overarching categories (figure 3). The first related to the *location* of provision; in an ideal service, physiotherapy services should span inpatient (28, 100%), outpatient (28, 100%) and community settings (15, 54%) (figure 3). Participants also reflected on the *role of the physiotherapist* within each of these settings. Inpatient physiotherapy was described as a high-intensity period of postoperative rehabilitation, with a focus on developmental or respiratory support. Outpatient service provision included standardised preoperative and postoperative monitoring, in particular, developmental surveillance, as well as ongoing rehabilitation. Provision of community services was portrayed as an adjunct to outpatient services, with tertiary centre hospitals providing education, outreach and clinical support alongside joint working.

There was variation from participants about who would provide this ‘ideal’ service—*workforce staffing*. Provision was defined by banding, by job title and whole-time equivalent staff numbers. All participants supported face-to-face service provision (*method*); however, there was a place for virtual support and advisory/consultation roles. When it came to defining who services should focus on (*service users*), there was wide variation, with definitions by age (under 5 years), ‘high risk’ individual patients, ‘high risk diagnoses’, patients screened as at risk and referral when issues identified. Reflective of this, there was a lack of consensus in duration and frequency of an ideal service provision. There was recognition from some that an ideal service would include *service evaluation/assessment of impact*, including validated assessment tools and outcome measures; see online supplemental material 4 for content analysis of the free-text question, including supporting quotes.

### Barriers

Physiotherapists reported a lack of funding as the primary barrier to service expansion (34, 100%). Commissioning of services (32, 94%), absence of guidelines (30, 88%) and inadequate staffing levels (28, 82%) were also viewed as important barriers. Seven (21%) physiotherapists reported geographical location as a barrier within free-text responses. 42 (91%) other HCPs reported funding as a barrier, followed by inadequate staffing (41, 89%). Among all respondents, volume of medical appointments (69, 86%), transportation (66, 83%) and finances (62, 78%) were reported as barriers to families attending outpatient appointments.

## Discussion

This study provides a unique insight into HCPs’ view on physiotherapy services and clinical practice in children with CHD in the UK and Ireland. There are currently no national standards or specifications for the provision of paediatric cardiac physiotherapy services^21^ to guide service delivery. Consequently, as demonstrated via this study, there is a lack of consensus regarding intervention frequency, duration, which patient groups should be prioritised, and the optimal timing of interventions. However, HCPs appreciated the value of delivering physiotherapy across multiple stages of the patient journey.

This study has demonstrated that assessment frequency in the UK and Ireland is comparable to other countries. In the USA, 45% of cardiologists complete developmental assessments,^22^ and in Australia, 54% of patients with a Fontan circulation undergo developmental evaluation.^23^ Given that children with CHD who experience functional impairments report lower QOL and reduced participation in physical activities,^24^,^26^ it is important to explore why half of UK and Ireland HCPs do not assess these children’s developmental or functional needs. A lack of specialist training and the time-consuming nature of developmental assessments are cited as the most common reasons why these critical assessments are not performed.^27 28^ The AHA statement suggests that children with CHD who fulfil high-risk criteria should automatically undergo developmental and functional assessments, which act as a gateway to onward referrals to therapists, including physiotherapists.^11^ Interestingly, our study found that HCPs are least likely to refer based on a high-risk criterion. This is consistent with data reported in the USA, where up to 90% of HCPs do not evaluate the risk of developmental delay in line with the AHA guidelines.^22^ We found that referrals to physiotherapists were primarily based on physical examination findings or because of parental concerns. A physical examination is not a standardised assessment and is often performed by personnel who are not specifically trained to assess for developmental and functional deficits. The non-standardised nature of this initial pathway to physiotherapy referral likely results in inadequate initial screening and missed referral opportunities. Similarly, relying on parental feedback can also lead to delayed referral, as many parents of children with CHD do not highlight their developmental concerns until their child reaches 3 years of age.^29^ The findings of this study suggest that developmental and functional evaluation plus referrals to physiotherapy are based on clinician preference and historical methods of evaluation. However, if medical staff do not have the appropriate training or time to complete developmental and functional evaluations, a significant number of children could be missed who would benefit from physiotherapeutic interventions. Consideration needs to be given as to how physiotherapists integrate into already established medical models of clinical practice.

Satisfaction with inpatient physiotherapy services varied in this study. Traditional roles of inpatient physiotherapists are changing due to the recognition that neonatal care and early rehabilitation and mobilisation are valuable.^30 31^ The difficulties encountered when trying to expand and adapt inpatient services were demonstrated by a UK and Ireland-based study, which reported 100% of sites completed a respiratory assessment in children with CHD following cardiac surgery, whereas only 10% routinely assessed motor delay or loss of function.^13^ This could explain why physiotherapists have differing views on inpatient service provision, as some hospitals may have made changes in service delivery, whereas others are struggling to adapt without additional resources. Physiotherapists identified clinical areas of importance with interventions provided on the ward post-surgery in children across all ages suggested as a priority. One study has examined the effects of physiotherapy provided in a ward environment for children under 3 years old following cardiac surgery. They found that physiotherapy interventions can aid in the recovery of lost motor skills.^32^ However, rehabilitation after a period of critical illness can be stressful for parents, whose main priority is the survival and comfort of their child.^33 34^ Therefore, understanding the feasibility, deliverability and effects of rehabilitation interventions administered in the immediate postoperative period should be considered when evaluating changes to clinical practice.

Physiotherapists highlighted the value of outpatient and community services, although negative views of existing community provision were common. Factors for this could include a lack of integration between acute and community services, prioritisation of children with neurological sequelae^35^ and prolonged waiting times (on average 21 weeks).^36^ The use of clinics, outpatient and cardiac rehabilitation programmes was suggested in this study to address continuity gaps between acute and community services. The value of physiotherapy outpatient rehabilitation on functional and developmental outcomes has been demonstrated.^37^,^39^ Furthermore, completion of cardiac rehabilitation programmes can improve QOL and cardiovascular fitness^40^ and has been evaluated as feasible and acceptable.^41^ However, clinical application is limited due to the heterogeneity of study populations and methodologies. To support clinical practice, further development and evaluation of outpatient interventions are needed.

The family and patient barriers highlighted in this study are consistent with multiple studies that demonstrate challenges with sustainability and acceptability of expanding cardiac services.^41^,^44^ HCPs highlighted a lack of funding as a significant barrier to changing service provision. Trusts must create services that are both clinically effective and financially sustainable. This is of particular significance when considering the expansion of outpatient physiotherapy services. The NHS Long Term Plan advocates for the expansion of community services and primary care over services developed in tertiary centres.^45^ Therefore, future outpatient care models must consider the financial and practical sustainability of services offered at specialist centres.

Limitations of this study include proportionately low response rates from specific professional groups. This limited subgroup analysis meant that individual professional groups had to be considered as a single group of non-physiotherapists. We only obtained the views of HCPs within level 1 paediatric cardiac surgical centres; therefore, it may not be representative of experiences elsewhere, including the community. Further exploration of individual professional views and wider HCP groups may be needed to support intervention development and delivery.

## Conclusions

HCPs appreciate the positive impact physiotherapy services can have on children with CHD and the value of service expansion. Physiotherapists highlighted the need for interventions throughout a child’s life, including neurodevelopmental interventions, postoperative rehabilitation and cardiac rehabilitation. However, further exploration is needed to understand the impact of physiotherapy interventions within each of these areas and what format of service delivery is optimal.

## Supplementary material

10.1136/bmjopen-2024-097314 online supplemental file 1

10.1136/bmjopen-2024-097314 online supplemental file 2

10.1136/bmjopen-2024-097314 online supplemental file 3

10.1136/bmjopen-2024-097314 online supplemental file 4

## Data Availability

All data relevant to the study are included in the article or uploaded as supplementary information.
