# Supplementary material for: Healthcare professionals’ views of physiotherapy after cardiac surgery in children with congenital heart disease: a UK and Ireland survey
Source: BMJ Open. 2025 Nov 12;15(11):e097314. doi: 10.1136/bmjopen-2024-097314 (PMC12612756; doi:10.1136/bmjopen-2024-097314)
Supplement: online supplemental file 1 [file bmjopen-15-11-s001.pdf]

Supplement 1. Literature to support survey question development.

Question	Literature
What is your profession?<br>Independent variable	n/a
At which Paediatric Congenital Cardiac Surgery centre in the UK or Ireland do you work?	n/a
How frequently do you discuss children's motor development or functional abilities with parents post cardiac surgery as part of your clinical role?	Marino et al, 2012; Ilardi et al, 2020 ; Ware et al, 2020; Bolduc et al, 2022; Eagleson et al, 2022; Williams et al, 2017
What would trigger you to consider a patient high-risk and require physiotherapy input post cardiac surgery?	Marino et al, 2012; Eagleson et al, 2022; Bolduc et al, 2022
For children with delayed motor development or reduced functional abilities following cardiac surgery, how satisfied are you with the inpatient physiotherapy service provision at your centre?	Lisanti et al, 2023; Rogers et al, 2023; Tikkanen et al, 2023
For children with delayed motor development or reduced functional abilities following cardiac surgery, how satisfied are you with the outpatient/community physiotherapy service provisions in your region?	Abell et al, 2023; Hoskote et al, 2020; Tikkanen et al, 2023
Based on your clinical experience how important is access to physiotherapy interventions in different age groups and stages of the patient journey to address delayed motor development or reduced functional abilities?	Ilardi et al, 2020; Tikkanen et al, 2023; Marino et al, 2012; Ware et al, 2020; Lisanti et al, 2023; Kendall et al, 2003; Bolduc et al, 2022
To ensure optimum access to space, equipment and accessibility for families, rank the following locations in order of importance where outpatient developmental physiotherapy interventions should be delivered in children post cardiac surgery	Tikkanen et al, 2023; Marino et al, 2012; Ware et al, 2020
Would expanding inpatient physiotherapy services to address delayed motor development or reduced functional abilities improve the care provided to children with CHD following cardiac surgery?	Tikkanen et al, 2023; Lisanti et al, 2023; Kendall et al, 2003

Question	Literature
Would expanding outpatient physiotherapy service provision to address delayed motor development or reduced functional abilities improve the care provided to children with CHD following cardiac surgery?	Tikkanen et al, 2023; Lisanti et al, 2023; Kendall et al, 2003
Do you think it is necessary for community/outpatient physiotherapists to have specialist experience and knowledge of congenital heart disease?	Marino et al, 2012; Tikkanen et al, 2023; Abell et al, 2023
Do you feel routine motor developmental screening during outpatient cardiology appointments by a physiotherapist would be of benefit to the patient and family?	Ilardi et al, 2020; Tikkanen et al, 2023; Marino et al, 2012
Describe an ideal physiotherapy service that addresses delayed motor development or reduced functional abilities in children with congenital heart disease. Consider service format, staffing, location, and frequency/ duration of interventions	Tikkanen et al, 2023; Clarke et al, 2023; Mitteregger et al, 2023
What would be the barriers to expanding physiotherapy services that address motor development and physical function in children with congenital heart disease post cardiac surgery in your centre?	Abell et al, 2023; Bolduc et al, 2022
What would be the barriers for families attending outpatient physiotherapy appointments at your centre?	Abell et al, 2023; Bolduc et al, 2022
