# Supplementary material for: Healthcare professionals’ views of physiotherapy after cardiac surgery in children with congenital heart disease: a UK and Ireland survey
Source: BMJ Open. 2025 Nov 12;15(11):e097314. doi: 10.1136/bmjopen-2024-097314 (PMC12612756; doi:10.1136/bmjopen-2024-097314)
Supplement: online supplemental file 2 [file bmjopen-15-11-s002.pdf]

Clarke SL et al. Healthcare professionals' views of physiotherapy after cardiac surgery.

Supplement 2. Face and content validation process

Face Validation

Item	Expert 1	Expert 2	Expert 3	Expert 4	Expert 5	Expert 6	Experts in agreement	Comments	Changes made
**Readability**: are the instruction in the survey adequate	N	Y	Y	Y	Y	Y	5	Introduction paragraphs needs to clarify post-surgical patients	Amended to include clarification on post-surgical patients
**Layout and style:** Do you think the format is appropriate?	Y	Y	Y	Y	Y	N	5	Needs bigger comment boxes	Comment boxes changes to larger size
Is the ordering of questions optimal?	Y	Y	Y	Y	N	Y	5	Move question 15 to after question 12	Altered order
**Clarity of wording:** Are the questions clear and unambiguous	N	Y	Y	Y	N	Y	4	See Table S2a	See Table S2a
**Feasibility:** Is the length of the survey appropriate	Y	Y	Y	Y	Y	Y	6	n/a	n/a

<u>Comments on wording clarity</u>

	Question	Comments	Changes made
3	For every child post-cardiac surgery in these age groups, how often do you discuss children's motor development or functional abilities with parents as part of your clinical role (ages 0-3, 4-7, 8-11, 12+)	Felt "how often" was too vague.	Wording altered to frequently
4	What would trigger you to consider referring a patient for physiotherapy input post-cardiac surgery?	Wording of 3rd response option felt not to be clear	Re-worded from reduction to reduced
5	How satisfied are you with the level of inpatient physiotherapy services available to children with delayed motor development or impaired physical function following cardiac surgery?	Felt too vague and unsure if they meant specific to their centre or not	Question wording altered
6	How satisfied are you with the level of outpatient physiotherapy services available to children with delayed motor development or impaired physical function following cardiac surgery?	As above<br>Add comments	Question wording altered
7	How important is access to physiotherapy interventions in different age groups and stages of the patient journey to address delayed motor development or reduced functional abilities? (ages 0-3, 4-7, 8-11, 12+) (post surgery PICU, post-surgery ward, post-surgery once discharged, pre-surgery)	Ordering of responses to include pre-surgery before surgery<br><br>Felt the clinical experience is important	Altered ordering of responses<br><br>Added context of clinical experience
8	For the following locations rank in order of importance where outpatient physiotherapy interventions should be delivered in children post-cardiac surgery (patient's home, hospital, community centre, school/ nursey)	Comments around what makes it important i.e space, equipment	Questions altered to improve clarification
9	Would expanding physiotherapy services to address motor development or	Felt this was too broad to answer and advised it was broken down into IP/OP	Question split into 2 separate questions

	reduction of physical function improve care provided to children with CHD following surgery		
10	Do you think it is necessary for community/outpatient physiotherapists to have experience and knowledge of congenital heart disease?	Add "specialist" to provide context	Question altered
11	Who is best placed to deliver outpatient physiotherapy	Professionals felt the question was vague and would not be answered clearly. Question answered via questions 8 and 10	Question removed
12	Do you feel routine motor developmental screening during outpatient cardiology appointments by a physiotherapist would be of benefit to the patient and family?		
13	What are the barriers to expanding physiotherapy services that address motor development and function for children with congenital heart disease post cardiac surgery?	Clarification around centre and potential barriers	Question altered
14	If services were expanded to deliver outpatient physiotherapy what in your opinion would the barriers be for families attending appointments?	Felt expansion of services not needed as barriers are set	Question altered
15	Describe an ideal physiotherapy service that addresses delayed motor development or reduced functional abilities in children with congenital heart disease.	Felt question too vague and suggested considerations for staff to follow	Considerations for staff added

Clarke SL et al. Healthcare professionals' views of physiotherapy after cardiac surgery.

Content Validation process

An I-CVI score above 0.83 and an average S-CVI above 0.8 were deemed acceptable to establish the validity of the items and instrument. Question 11 did not achieve the recommended I-CVI score and therefore was removed from the survey.

Item	Expert 1	Expert 2	Expert 3	Expert 4	Expert 5	Expert 6	Experts in agreement	I-CVI
Q1	1	1	1	1	1	1	6	1
Q2	1	1	1	1	1	1	6	1
Q3	1	1	1	1	1	1	6	1
Q4	1	1	1	1	1	1	6	1
Q5	1	1	1	1	1	1	6	1
Q6	1	1	1	1	1	1	6	1
Q7	1	1	1	1	1	1	6	1
Q8	0	1	1	1	1	1	5	0.83
Q9	1	1	1	1	1	1	6	1
Q10	1	1	1	1	1	1	5	1
Q11	0	1	1	0	1	1	4	0.6
Q12	1	1	1	1	1	1	6	1
Q13	1	1	1	1	1	1	6	1
Q14	1	1	1	1	1	1	6	1
Q15	1	1	1	1	1	1	6	1
Q16	1	1	1	1	1	1	6	1
							S-CVI/Avg	0.96

S-CVI/ AVG- Average Scale Content Validity Index; I-CVI- Item Content Validity Index.

Shi J, Mo X, Sun Z. Content validity index in scale development. Journal of Central South University Medical sciences 2012;37(2):152-5.
