# Supplementary material for: Healthcare professionals’ views of physiotherapy after cardiac surgery in children with congenital heart disease: a UK and Ireland survey
Source: BMJ Open. 2025 Nov 12;15(11):e097314. doi: 10.1136/bmjopen-2024-097314 (PMC12612756; doi:10.1136/bmjopen-2024-097314)
Supplement: online supplemental file 3 [file bmjopen-15-11-s003.pdf]

Healthcare professionals' views on physiotherapy services and interventions in children following cardiac surgery: a UK and Ireland survey

We are conducting this survey to establish healthcare professionals' views on physiotherapy interventions and services that address delayed motor development or impaired functional abilities in children with congenital heart diseases following cardiac surgery in the UK & Ireland.Delayed motor development occurs when a child is not meeting expected motor milestones for their age. Impaired functional abilities encompass physical symptoms that limit participation in childhood activities and can impact the development of motor skills.

This survey will take approximately 5-10 minutes to complete. You may save & return later by clicking the button at the end of this survey, then follow the link in the email or click 'Returning?' at the top right of this page and enter your individual return code.

The survey will close on Wednesday 31st July 2024. Your participation is entirely voluntary and you can withdraw at any time by emailing: sxc402@student.bham.ac.uk. You can find out what we do with your data in our Privacy Notice.

What is your professional role?

- ◯ Consultant Paediatric Cardiac Surgeon
- ◯ Consultant Paediatric Cardiologist
- ◯ Cardiac Nurse Specialist
- ◯ Paediatric Physiotherapist
- ◯ Paediatric Occupational Therapist
- ◯ Other

Please specify "Other"

____

At which paediatric cardiac surgery centre do you work?

- ◯ Birmingham, Children's Hospital
- ◯ Bristol, Royal Hospital for Children
- ◯ Dublin, Our Lady's Children's Hospital
- ◯ Glasgow, Royal Hospital for Children
- ◯ Leeds, General Infirmary
- ◯ Leicester, Glenfield Hospital
- ◯ Liverpool, Alder Hey Children's Hospital
- ◯ London, Evelina London Children's Hospital
- ◯ London, Great Ormond Street Hospital
- ◯ London, Royal Brompton Hospital
- ◯ Newcastle, Freeman Hospital
- ◯ Southampton, University Hospital

For every child post-cardiac surgery in these age groups, how frequently do you discuss children's motor development or functional abilities with parents as part of your clinical role? [select one per age group]

	Never	Rarely	Sometimes	Usually	Always	Not part of role
0-3 years	◯	◯	◯	◯	◯	◯

4-7 years	◯	◯	◯	◯	◯	◯
8-11 years	◯	◯	◯	◯	◯	◯
12+ years	◯	◯	◯	◯	◯	◯

What would trigger you to consider referring a patient for physiotherapy input post cardiac surgery? [select all that apply]

- ☐ Meets high-risk criteria i.e ECMO, cardiopulmonary resuscitation
- ☐ Signs of developmental delay or reduced functional ability on formal examination
- ☐ Parental concerns of delayed development or reduced functional ability
- ☐ Nil / not applicable to my role

	Very dissatisfied	Dissatisfied	Neither satisfied nor dissatisfied	Satisfied	Very satisfied	No opinion
For children with delayed motor development or impaired functional abilities following cardiac surgery, how satisfied are you with the inpatient physiotherapy service provision at your centre? [select one from list]	◯	◯	◯	◯	◯	◯

	Very dissatisfied	Dissatisfied	Neither satisfied nor dissatisfied	Satisfied	Very satisfied	No opinion
For children with delayed motor development or impaired functional abilities following cardiac surgery, how satisfied are you with the outpatient/community physiotherapy service provisions in your region? [select one from list]	◯	◯	◯	◯	◯	◯

Based on your clinical experience, how important is access to physiotherapy interventions in different age groups and stages of the patient journey to address delayed motor development or reduced functional abilities? [select one per category]

	Not important	Low importance	Somewhat important	High importance	Very high importance	No opinion
0-3 years	◯	◯	◯	◯	◯	◯
4-7 years	◯	◯	◯	◯	◯	◯
8-11 years	◯	◯	◯	◯	◯	◯
12+ years	◯	◯	◯	◯	◯	◯
Pre surgery	◯	◯	◯	◯	◯	◯

Post surgery PICU/CICU	◯	◯	◯	◯	◯	◯
Post surgery on the ward	◯	◯	◯	◯	◯	◯
Post surgery once discharged home	◯	◯	◯	◯	◯	◯

To ensure optimum access to space, equipment and accessibility for families, please rank the following locations in order of importance where outpatient physiotherapy interventions should be delivered in children post cardiac surgery. [rank by selecting one option per column]

	Least important	Less important	Middle importance	More important	Most important
Within the patient's home	◯	◯	◯	◯	◯
Within the hospital	◯	◯	◯	◯	◯
Within a community healthcare centre	◯	◯	◯	◯	◯
School/ nursery	◯	◯	◯	◯	◯
Virtually	◯	◯	◯	◯	◯

The following questions relate to future developments in physiotherapy services for children with CHD.

Would expanding inpatient physiotherapy service provision to address delayed motor development or reduced functional abilities improve the care provided to children with CHD following cardiac surgery?

◯ Yes
◯ No
◯ Unsure

Would expanding outpatient physiotherapy service provision to address delayed motor development or reduced functional abilities improve the care provided to children with CHD following cardiac surgery?

◯ Yes
◯ No
◯ Unsure

Do you think it is necessary for community/outpatient physiotherapists to have specialist experience and knowledge of congenital heart disease?

◯ Yes
◯ No
◯ Unsure

Do you feel routine motor developmental screening during outpatient cardiology appointments by a physiotherapist would be of benefit to the patient and family?

◯ Yes
◯ No
◯ Unsure

Describe an ideal physiotherapy service that addresses delayed motor development or reduced functional abilities in children with congenital heart disease. Consider service format, staffing, location, and frequency/ duration of interventions.

____

What would be the barriers to expanding physiotherapy services that address delayed motor development or reduced functional abilities for children with congenital heart disease post cardiac surgery in your centre? [select all that apply]	☐ Lack of dedicated funding<br>☐ Lack of national guidelines supporting service provision<br>☐ Lack of staffing to provide service<br>☐ Lack of space to deliver therapy<br>☐ Lack of commissioned service<br>☐ Lack of service need<br>☐ I don't perceive there are any barriers<br>☐ Other
Please specify "Other"	__________________________________
What would be the barriers for families attending outpatient physiotherapy appointments at your centre? [select all that apply]	☐ Childcare commitments<br>☐ Burden of medical appointments<br>☐ Employer obligations<br>☐ Child's academic attainment<br>☐ Transportation issues<br>☐ Financial issues<br>☐ I don't perceive there are any barriers<br>☐ Other
Please specify "Other"	__________________________________

The findings of this survey will be used to inform the design of future research, including physiotherapy interventions that can be implemented into clinical practice and accessed by families.

If you are happy for us to contact you regarding future research, please provide your name and email address below.

Name:	__________________________________
Email address:	__________________________________
