# Supplementary material for: Healthcare professionals’ views of physiotherapy after cardiac surgery in children with congenital heart disease: a UK and Ireland survey
Source: BMJ Open. 2025 Nov 12;15(11):e097314. doi: 10.1136/bmjopen-2024-097314 (PMC12612756; doi:10.1136/bmjopen-2024-097314)
Supplement: online supplemental file 4 [file bmjopen-15-11-s004.pdf]

Supplement 4. Content analysis including categories, subcategories and supporting quotes.

Category	Subcategory n (%)	Quote
Location	Inpatient 28 (100)	"Assessment as an inpatient, ongoing early intervention post-surgery" (participant 9)
	Outpatient 28 (100)	referrals and intervention as an outpatient as required once identified (participant 20)
	Community 15 (54)	referral to community physiotherapy services if required at point of discharge home (participant 27)
Physiotherapist role	Developmental assessment (all 3 locations) 6 (21)	"To have physios in follow up clinics to monitor neurodevelopment with standardised assessments" (participant 24)
	Screening (all 3 locations) 6 (21)	"Preadmission screening and baseline with appropriate outcome measure" (participant 17)
	Developmental input (inpatient/outpatient) 6 (21)	Neurodevelopmental team with dedicated hours to cardiac inpatients to support the long stay cardiac babies development (participant 10)
	Rehabilitation (inpatient/outpatient) 6 (21)	"Cardiac rehab and intervention post admission to ensure back to baseline and education re future activities" (participant 18)
	Referral (all 3 locations) 6 (21)	Ability for MDT to refer patients from clinic if concerns at different stage in pathway (participant 12)
	Communication with MDT (all 3 locations) 6 (21)	"Relies on strong communication links between community and acute therapy teams" (participant 13)
	Education (all 3 locations) 6 (21)	"Outreach and education from the hospital setting to support community teams" (participant 10)

	Outreach (community) 1 (3)	"scope to complete home visits as required" (participant 15)
Method of involvement	Face to face 6 (21)	Face to face initially, then could be virtual follow up (participant 18)
	Virtual 4 (14)	Depending on the goal of the course of physio, may be more beneficial to provide a couple of face to face appointments to obtain better assessment data then continue with virtual follow ups (participant 27)
	Advisory 4 (14)	Community follow up with specialist CHD guidance/advice as needed (participant 4)
Workforce staffing	Title 16 (57)	Specialist Paediatric Cardiology Physiotherapist (participant 3)
	Standalone clinic 10 (36)	standalone cardiac/cardiology physiotherapy clinic to address physiotherapy specific issues related to cardiac disease (participant 3)
	MDT clinic 17 (61)	physio follow up in MDT cardiac clinic (participant 13)
	Banding 4 (14)	Solely focused band 7 physio, devoted to cardiac pts on PICU, f/u from ECMO, ward and post op clinics for f/u (participant7)
Service users	All CHD patients 14 (50)	Screening assessment via parental questionnaire for all CHD children to determine if a more in-depth assessment is required (participant 8)
	Children with known delay 7 (25)	An annual assessment for children with known motor delays (participant 8)
	Diagnosis criteria/ "high risk" criteria 5 (33)	Format of the course of physio would likely be condition specific as well as patient specific, e.g. people with some conditions may need more input acutely

		than others, some may need longer follow up, and some congenital conditions would require much earlier interventions than others e.g. some valve replacements vs Norwood procedure would be provided at different ages (participant 27)
	Referrals 2 (7)	Ability for MDT to refer patients from clinic if concerns at different stage in pathway (participant 12)
	Age criteria <5yrs 2 (7)	A service dedicated to under 5s undergoing/underwent cardiac surgery (participant 28)
Service evaluation/impact	Standardised assessment 3 (20)	Standardised neuro-dev assessments e.g. Bayley 4 (participant 9)
	Broder interventions 2 (7)	School interventions with SENCO and ed psych (participant 9)
