# Supplementary material for: Healthcare professionals’ views of physiotherapy after cardiac surgery in children with congenital heart disease: a UK and Ireland survey
Source: BMJ Open. 2025 Nov 12;15(11):e097314. doi: 10.1136/bmjopen-2024-097314 (PMC12612756; doi:10.1136/bmjopen-2024-097314)
Supplement: Supplementary file 5 [file bmjopen-2024-097314.reviewer_comments.pdf]

PEER REVIEW HISTORY

BMJ Open publishes all reviews undertaken for accepted manuscripts. Reviewers are asked to complete a checklist review form and are provided with free text boxes to elaborate on their assessment. These free text comments are reproduced below.

ARTICLE DETAILS

Title (Provisional)

Healthcare professionals' views of physiotherapy after cardiac surgery in children with congenital heart disease: a UK and Ireland survey

Authors

clarke, stephanie; Shkurka, Emma; Menzies, Julie C.; Drury, Nigel E

VERSION 1 - REVIEW

**Reviewer**	**1**
**Name**	**Hullumani, Sharath**
**Affiliation**	**Datta Meghe Institute of Higher Education and Research Deemed to be University, paedaitrics Physiotherapy**
**Date**	**17-Feb-2025**
**COI**	**None**

Objectives are not clear, but research question were clear.

**Reviewer**	**2**
**Name**	**Adreak, Najah**
**Affiliation**	**The University of British Columbia, Surgery**
**Date**	**02-Jun-2025**
**COI**	**None**

Submitted on behalf of reviewer.

**Reviewer**	**3**
**Name**	**Eisenga, John**
**Affiliation**	**Department of Research, Baylor Scott and White Research Institute Plano, Texas.**
**Date**	**15-Sep-2025**

**COI**	**None**

Clarke and colleagues present an interesting study assessing the views of healthcare professionals of physiotherapy following cardiac surgery in patients with congenital heart disease. This is an interesting study which will likely have positive impacts in improving longitudinal care in this population by trying to assess the benefits and barriers of this treatment. Importantly, this survey highlighted the perceived benefits of these treatments while attempting to better understand the barriers from a healthcare providers point of view. I believe this is a paper worthy of publication on a timely subject. I would like to commend the authors for this undertaking and would like to see future studies addressing how these barriers may be overcome.

VERSION 1 - AUTHOR RESPONSE

Reviewer: 1

Dr. Sharath Hullumani, Datta Meghe Institute of Higher Education and Research Deemed to be University, Ravi Nair Physiotherapy College

Comments to the Author:

Objectives are not clear, but research question were clear.

Reviewer: 2

Dr. Najah Adreak, The University of British Columbia

Comments to the Author:

Submitted on behalf of reviewer.

Reviewer: 3

Dr. John Eisenga, Department of Research, Baylor Scott and White Research Institute Plano, Texas.

Comments to the Author:

Clarke and colleagues present an interesting study assessing the views of healthcare professionals of physiotherapy following cardiac surgery in patients with congenital heart disease. This is an interesting study which will likely have positive impacts in improving longitudinal care in this population by trying to assess the benefits and barriers of this treatment. Importantly, this survey highlighted the perceived benefits of these treatments while attempting to better understand the barriers from a healthcare providers point of view. I believe this is a paper worthy of publication on a timely subject. I would like to commend the authors for this undertaking and would like to see future studies addressing how these barriers may be overcome.

Reviewer: 1

If you have selected ‘Yes’ above, please provide details of any competing interests.: Not applicable

Reviewer: 2

If you have selected ‘Yes’ above, please provide details of any competing interests.: Not applicable.

Reviewer: 3

If you have selected ‘Yes’ above, please provide details of any competing interests.: not applicable
